# Cerebrospinal Fluid from Patients with Subarachnoid Haemorrhage and Vasospasm Enhances Endothelin Contraction in Rat Cerebral Arteries

**DOI:** 10.1371/journal.pone.0116456

**Published:** 2015-01-28

**Authors:** Barbara Assenzio, Erica L. Martin, Edgaras Stankevicius, Federica Civiletti, Marco Fontanella, Riccardo Boccaletti, Maurizio Berardino, AnnaTeresa Mazzeo, Alessandro Ducati, Ulf Simonsen, Luciana Mascia

**Affiliations:** 1 Department of Anesthesia and Intensive Care, Azienda Ospedaliera Città della salute e della scienza di Torino, University of Turin, Turin, Italy; 2 Institute of Physiology and Pharmacology, Medical Academy, Lithuanian University of Health Sciences, Kaunas, Lithuania; 3 Division of Neurosurgery, Department of Neuroscience, Azienda Ospedaliera Città della salute e della scienza di Torino, University of Turin, Turin, Italy; 4 Department of Biomedicine, Pulmonary and Cardiovascular Pharmacology, University of Aarhus, Aarhus, Denmark; University of Southampton, UNITED KINGDOM

## Abstract

**Introduction:**

Previous studies have suggested that cerebrospinal fluid from patients with subarachnoid hemorrhage (SAH) leads to pronounced vasoconstriction in isolated arteries. We hypothesized that only cerebrospinal fluid from SAH patients with vasospasm would produce an enhanced contractile response to endothelin-1 in rat cerebral arteries, involving both endothelin ET_A_ and ET_B_ receptors.

**Methods:**

Intact rat basilar arteries were incubated for 24 hours with cerebrospinal fluid from 1) SAH patients with vasospasm, 2) SAH patients without vasospasm, and 3) control patients. Arterial segments with and without endothelium were mounted in myographs and concentration-response curves for endothelin-1 were constructed in the absence and presence of selective and combined ET_A_ and ET_B_ receptor antagonists. Endothelin concentrations in culture medium and receptor expression were measured.

**Results:**

Compared to the other groups, the following was observed in arteries exposed to cerebrospinal fluid from patients with vasospasm: 1) larger contractions at lower endothelin concentrations (p<0.05); 2) the increased endothelin contraction was absent in arteries without endothelium; 3) higher levels of endothelin secretion in the culture medium (p<0.05); 4) there was expression of ET_A_ receptors and new expression of ET_B_ receptors was apparent; 5) reduction in the enhanced response to endothelin after ET_B_ blockade in the low range and after ET_A_ blockade in the high range of endothelin concentrations; 6) after combined ET_A_ and ET_B_ blockade a complete inhibition of endothelin contraction was observed.

**Conclusions:**

Our experimental findings showed that in intact rat basilar arteries exposed to cerebrospinal fluid from patients with vasospasm endothelin contraction was enhanced in an endothelium-dependent manner and was blocked by combined ET_A_ and ET_B_ receptor antagonism. Therefore we suggest that combined blockade of both receptors may play a role in counteracting vasospasm in patients with SAH.

## Introduction

Cerebral vasospasm is one of the most serious complications following aneurysmal subarachnoid hemorrhage (SAH) and is independently associated with poor outcome [1]. Endothelin-1 (ET-1) is thought to be of major importance for cerebral vasospasm [2,3]

In experimental models of SAH, increased sensitivity of cerebral arteries to ET-1 have been invariably reported [4]; increased levels of ET_A_ receptor mRNA have been inconsistently demonstrated [5] [6,7]while increased levels of ET_B_ mRNA have been unequivocally reported [8–10] [11]. So far studies on endothelin-1 receptor regulation did not differentiate between the two conditions of SAH with and without vasospasm [6,9,10,12]. Moreover, and *in vitro* studies on the cerebral microvasculature have often been used to explain events observed in conductive vessels[13]. Furthermore, although the cerebral endothelium is unique in terms of growth and responsiveness to various vasoactive agonists, results obtained in non-cerebral endothelial cells have been extrapolated to the brain vasculature[14,15].

The efficacy of selective receptor antagonists have been tested in experimental models of cerebral vasospasm [5,16,17]. Several randomized clinical trials tested the efficacy of ET_A_ selective blockade and found a reduction in angiographic vasospasm but no improvement in measureable functional outcomes [18–22]. These results pose several questions: 1) the possibility that early brain injury occurring just after the hemorrhage may contribute to the poor outcome; 2) a functional interaction between ET_A_ and ET_B_ receptors named “cross talk” may play a role in the pathogenesis of SAH-induced vasospasm.

To overcome the above-mentioned methodological criticisms and to elucidate the functional interaction between the two receptors, we evaluated the upregulation of the endothelin system of the targeted vessels by vasospasm, incubating intact and denuded conductive cerebral vessels with CSF from SAH patients with a conclusive diagnosis of vasospasm obtained by angiography.

We hypothesized that CSF from SAH patients who developed vasospasm would produce an enhanced contractile response in intact rat cerebral arteries involving both ET_A_ and ET_B_ receptors.

## Materials and Methods

### Patient Selection

#### Ethical approval

The present study was conducted using CSF from patients with aneurysmal SAH confirmed by CT scan and angiography admitted to ICU. CSF had been collected for an observational study in SAH patients at risk of developing vasospasm (NCT01686763). The institutional review board (Comitato Etico Interaziendale A.O.U. San Giovanni Battista di Torino A.O. C.T.O./Maria Adelaide di Torino) approved the study. If the patient was unable to give consent at study entry, consent was delayed, and the family was informed of the study. Written permission for using collected data was thus obtained from the patient (if competent) or from the family (in case of death or if the patient remained incompetent).

All the patients fulfilled the following inclusion criteria: 1) angiographic proof of aneurysm; 2) admission within 24 hours of the SAH; and 3) presence of an intraventricular catheter placed either after admission or at the time of surgery. Patients were graded clinically according to the World Federation of Neurological Surgeons (WFNS) scale and classified according to the CT distribution of blood as described by the Fisher scale.

All patients underwent neurosurgical intervention or endovascular procedure to secure the aneurysm within 2 days of admission. Neurological status was evaluated daily using the Glasgow Coma Scale (GCS), transcranial Doppler of the middle cerebral arteries was performed daily until day 14 and patients were treated according to the guidelines [1].

On day 7 post-SAH, a second angiogram was routinely performed and the patients were then classified as having: 1) angiographic vasospasm (AV) if the angiogram showed 25% or more reduction from baseline caliber without any clinical deterioration; 2) **clinical vasospasm (CV)** if a reduction in the angiographic caliber >50% of the middle cerebral artery (MCA) was accompanied by controlateral weakness to or global neurological deterioration (two points reduction in GCS) occurring later than day 3 for anterior or diffuse vasospasm; 3) **patients without vasospasm (NV)** if there was reduction lower than 25% in the angiographic caliber [23] [24].

To test the hypothesis of the present study samples of CSF from patients with the most severe vasospasm (**clinical vasospasm CV**) and from those without **vasospasm (no vasospasm NV)** were included. As **negative control** a group of patients with normal pressure hydrocephalus (NPI) and normal GCS undergoing lumbar drainage for the diagnosis of NPI was included [25]. In SAH patients CSF was collected daily until day 7, with the samples collected on days 4–5 used for the present study. CSF was drawn into tubes containing EDTA from the intraventricular catheter and collected by ventricular drainage from hydrocephalus patients. Tubes were kept and transported in ice. All CSF samples were centrifuged at 1700g for 15 min at 4°C to remove intact cells and cellular debris, following which the supernatant was frozen at −80°C.

### Functional study of the *Ex Vivo* Model of SAH-induced Vasospasm

Isolated rat basilar arteries were obtained from male Wistar rats (350–400g) (Charles River Laboratories, Calco, Italy). Rats were group-housed, had free access to standard chow and water in a controlled facility providing a 12-hour light/dark cycle, and were kept according to institutional animal welfare guidelines and legislation, submitted and approved by the local Animal Ethics Committee (Comitato di Bioetica dell’Ateneo dell’Università degli Studi di Torino) and all efforts were made to minimize suffering. Rats were sacrificed by carbon dioxide overdose followed by cervical dislocation and the cerebral basilar artery was isolated using a stereomicroscope and immersed in ice-cold physiological salt solution (PSS) (CaCl_2_ 2H_2_O 2.5 mmol/L, NaCl 119 mmol/L, KCl 4.69 mmol/L, glucose 5.5 mmol/L, MgSO_4_ 7H_2_O 1.17nmol/L, NaHCO_3_ 25 mmol/L, KH_2_PO_4_ 1.18 mmol/L and ethylenediaminetetraacetic (EDTA) 0.03 mmol/L). Segments of approximately 2 mm in length were incubated with 5% human CSF (v/v) obtained from ventricular drainage of the patients included in the study. Incubation lasted 24 hours at 37°C with 5% CO_2_ in O_2_ in Dulbecco’s modified Eagle’s medium (DMEM). The culture medium was then collected and frozen at −80°C for analysis of ET-1 concentrations.

Following incubation with human CSF, isolated rat basilar arteries were mounted on 40 μm tungsten wires in a small vessel myograph (D.M.T., Aarhus, Denmark) for isometric force recording [26]. Measurements were recorded using the ICU-Lab software program (KleisTEK Advanced Electronic Systems; Bari, Italy). The vessels were allowed to equilibrate for about 30 min in PSS, which was continuously aerated with oxygen enriched with 5% CO_2_ to maintain a pH of 7.4. The relation between resting wall tension and internal circumference was determined, and the internal circumference, L_100_, corresponding to a transmural pressure of 100 mmHg for a relaxed vessel in situ was calculated. The vessels were set to the internal circumference L_1_, given by L_1_ = 0.9L_100_. The effective internal lumen diameter was determined as *l*_1_ = L_1_/π, and was 306±41 mm (mean±SD, N = 106) and 303±38 μm (mean±SD, N = 20) in vessels with and without endothelium respectively.

After a 30 min period to stabilize vessel tone, the contractile capacity was determined by exposing the vessels to an isotonic solution containing 123 mmol/l of K^+^ (KCl 123.70 mmol/L, MgSO_4_ 7H_2_O 1.17 nmol/L, KH_2_PO_4_ 1.18 mmol/L, CaCl_2_ 2H_2_O 2.5 mmol/L, NaHCO_3_ 25 mmol/L, EDTA 0.03 mmol/L, glucose 5.5 mmol/L), obtained by equimolar change of NaCl for KCl in PSS. Only vessels responding by contraction corresponding to a pressure above 13.3 kPa to potassium were included in the study. The presence of an intact endothelium was checked by contracting the vessel using serotonin (5-HT, 10^−5^ mol/L, Sigma-Aldrich, Milan, Italy) and subsequently exposing the segments to histamine (10^−5^ mol/L, Sigma-Aldrich, Milan, Italy) to stimulate endothelium-dependent relaxation. Confirmation of the presence of endothelium was determined by a relaxation >50% (E^+^). In a separate set of experiments the endothelial cell layer was mechanically removed by gentle rubbing the intimal surface with a human hair (E^−^). After endothelial cells removal the contractility of the vessels was tested by adding serotonin.

We did not find any difference in serotonin response between groups with or without endothelial cells. The response to 5-HT was 1.59±0.56 (n = 6) and 1.43±0.48 mN/mm (n = 8), respectively, in vessels with and without endothelium exposed to CSF from the Control group, and 1.59±0.56 (n = 6) and 1.34±0.44 mN/mm (n = 6) in vessels with and without endothelium exposed to CSF from the Non-Vasospasm group, and 1.79±0.50 (n = 6) and 1.74±0.43 mN/mm (n = 6) in vessels with and without endothelium exposed to CSF from the Vasospasm group.

In a separate set of vessels, following exposure of the isolated basilar arteries to 5% human CSF, vessels were incubated with specific antagonists to 1) ET_A_ (BQ-123, 1×10^−5^ mol/L, Sigma-Aldrich, Milan, Italy), 2) ET_B_ (BQ-788, 1×10^−6^ mol/L, Sigma-Aldrich S.r.l, Milan, Italy) 3) both ET_A_ and ET_B_ (BQ-123 and BQ-788) 4) vehicle control for 30 min prior to performing concentration-response curves by cumulative application of ET-1.

### Enzyme-linked Immunosorbent Assay (ELISA)

ET-1 concentrations in CSF and in culture medium obtained before and after the incubation of the arteries were determined by ELISA (Human ET-1 (1–21), with sensitivity <0.05 pg/ml; Biomedica Diagnostics Inc, Vienna, Austria).

### Immunofluorescence

After the 24-hour incubation in CSF, basilar arteries were embedded in Tissue-Tek (Sakura, Zoeterwoude, NL) and frozen in liquid nitrogen. Cross-sections of the embedded vessels were then cut into 20 μm sections. The first antibodies used were rabbit anti-rat ET_A_ (AB3260, Chemicon International, Vimodrone, Italy), diluted 1:200, and rabbit anti-rat ET_B_ (AB3284, Chemicon International, Vimodrone, Italy), diluted 1:200 or irrelevant isotype contol antibody. All dilutions were performed in PBS with 5% bovine serum albumin (Sigma-Aldrich., Milan, Italy). The second antibodies used were goat anti-rabbit Alexafluor 488 conjugated (A11070, Invitrogen, San Giuliano Milanese, Italy), diluted 1:500 in PBS with 5% bovine serum albumin. The antibodies were detected at the appropriate wavelength using confocal microscopy (Leica TCS SP2, Mannheim, Germany). As control, only secondary antibodies were used. All samples were scanned in the same parameter setting throughout each series of immunoincubation, including same size of pin hole, gain level, black level, and laser power. The quantification analysis of immunoreactivity was carried out on single confocal image using Leica confocal software. As first step, background fluorescence was estimated by analysing the distribution of the pixel intensities in the image areas that did not contain any immunoreactive objects (the background threshold). The background was subtracted by setting the baseline of pixel intensities to the background value; autofluorescence was then subtracted. In the next step, an arbitrarily outlined polygon, which covered the vessel-occupied and imaged area, was chosen. In the polygon area, the relative area of immunolabelled pixels with an intensity value above the background was calculated. The fluorescence in five different areas on each artery was measured and the mean value for each vessel was used.

### Statistical Analysis

#### Data analysis

All recorded data were analyzed and expressed as tension (mN/mm) developed by the artery, defined as the force induced divided by two times the vessel length because we did not find any differences on potassium induced contraction among groups. In a given experiment, E_max_ denotes the maximal contractile response, and pEC_50_ denotes the negative logarithm of the concentration that elicits one-half of the maximal response. For biphasic responses, E_max1_ and pEC_50(1)_ were used to describe the high-affinity phase and E_max2_ and pEC_50(2)_ were used to describe the low-affinity phase of the concentration-response curves.

Continuous data are presented as mean ± standard error of the mean. Data normally distributed were analyzed by one-way or two-way ANOVA, followed by a Student’s t-test or Tukey-Kramer post-hoc test where appropriate, and data not normally distributed were analyzed by a non-parametric Kruskal-Wallis with Dunn’s post-hoc test. Differences were considered significant when the probability value was <0.05. Statistical analysis was performed using SPSS-statistical software, version 17.0 (SPSS Inc. Chicago, IL).

## Results

Inclusion criteria, demographic and clinical data of patients with subarachnoid hemorrhage included in the present study are reported in Table 1. The control group consisted of six patients (4 females, mean age 54±10) with diagnosed communicating hydrocephalus and normal GCS undergoing lumbar drainage.

**Table 1:** Demographic and clinical data of 17 patients studied.

	**SAH Non-Vasospasm (8)**	**SAH Vasospasm (9)**
Gender (M/F)	3/5	4/5
Age (years)	61 ± 11	57 ± 12
Aneurysm location (n)		
MCA	2	4
A com	4	2
ICA	1	1
Basilar	1	1
WFNS grade I-II (n)	3	3
WFNS grade III-V (n)	5	6
clipping/coiling	7/1	8/1
GCS admission	11±3	11±3
Fisher grade 3 (n)	4	4
Fisher grade 4 (n)	4	5

Data expressed as mean ± SD or N; MCA = middle cerebral artery, ICA = internal carotid artery, A com = anterior communicating WFNS = World federation of neurological surgeons, GCS = Glasgow Coma Scale

### Functional Analysis

**Figure 1:**
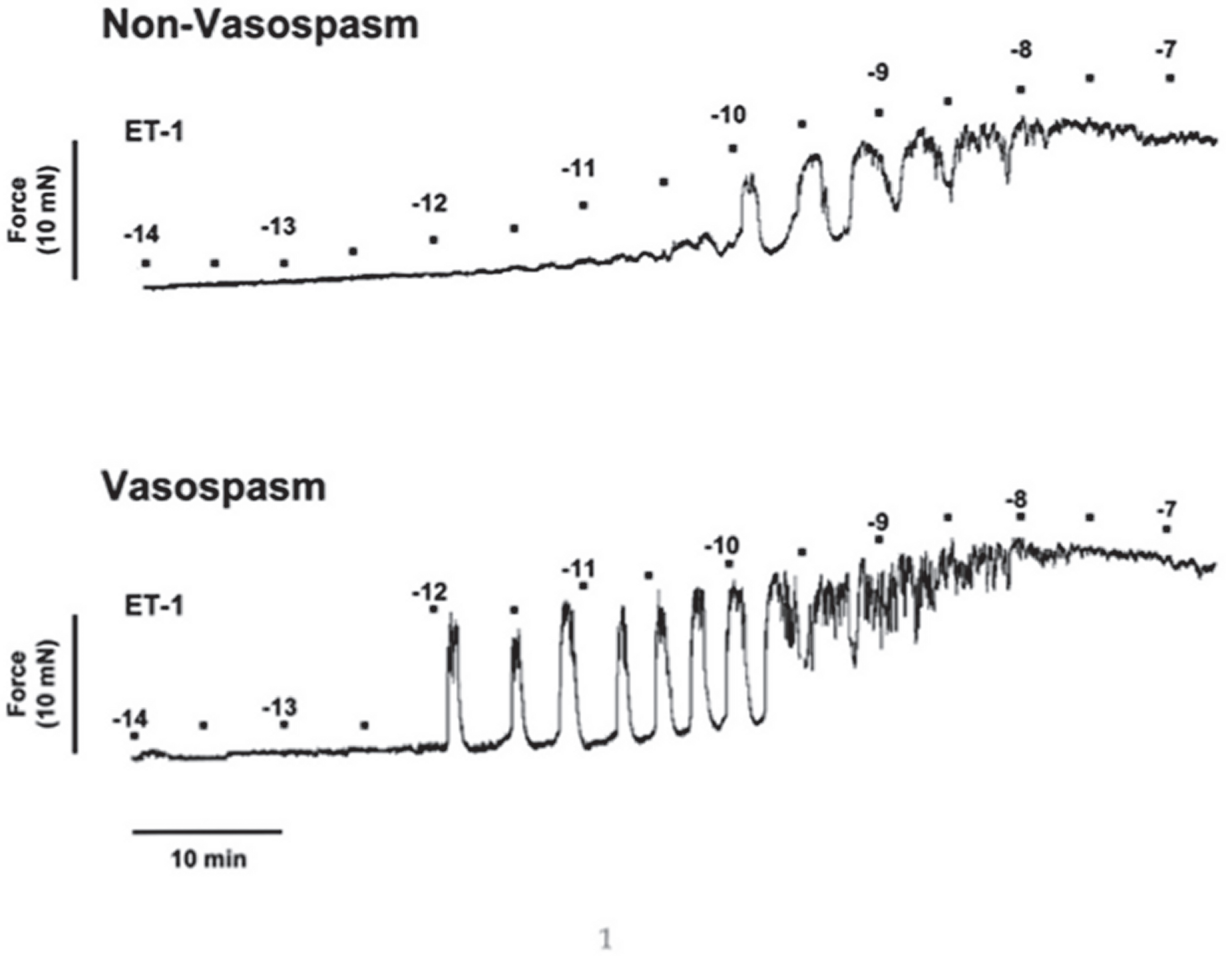
Experimental myograph record of isolated rat basilar arteries incubated with 5% CSF from patients with SAH with and without vasospasm. The contractile force generated during exposure to increasing doses of ET-1 by Log (0.5 mol/L) every 5 min was higher at lower ET-1 concentrationsfor ***Vasospasm*** (bottom panel) compared to ***Non-Vasospasm*** (top panel).

Rat basilar arteries incubated with CSF from ***Vasospasm*** patients developed greater contractile forces at lower ET-1 concentrations compared to vessels incubated with ***Non-Vasospasm*** CSF, as shown in the representative myograph trace (Fig. 1).

#### Experiments in arteries with endothelium

**Figure 2:**
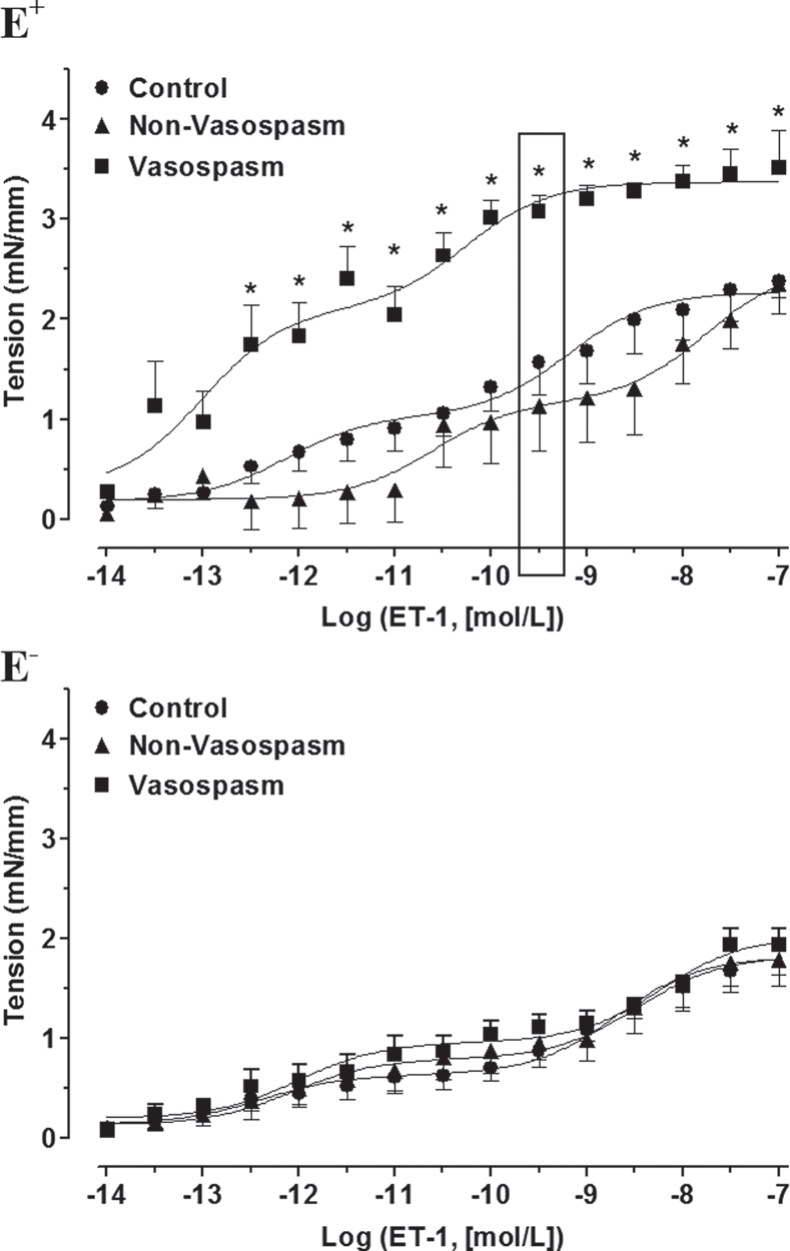
Concentration-response curves elicited by ET-1 in isolated rat basilar arteries with endothelium (E^+^) and without enodthelium (E^−^) following incubation with 5% human CSF from *Control* (n = 6), *Non-Vasospasm* (n = 6) and *Vasospasm* (n = 6) patients (upper panel) and from *Control* (n = 8), *Non-Vasospasm* (n = 6) and *Vasospasm* (n = 6) patients (lower panel). The response to ET-1 was significantly increased in the ***Vasospasm*** group compared to ***Control*** and ***Non-Vasospasm*** groups only in intact vessels. Significance by Kruskal Wallis with Dunn’s post-hoc test where *p<0.05 = ***Vasospasm*** *versus* ***Control*** and ***Non-Vasospasm***.

In E^+^ vessels an enhanced concentration-dependent contractile response to ET-1 was present in the ***Vasospasm*** group when compared with the ***Non-Vasospasm*** and ***Control*** groups at ET-1 concentrations between 1×10^−12.5^ and 1×10^−7^ mol/L (p<0.05) (Fig. 2 upper panel). Interpolation of the concentration-response curves showed a biphasic response in all three groups. Analysis of the E_max_ and pEC_50_ for each phase showed that the E_max1_, E_max2_ and pEC_50(2)_ were significantly higher in the ***Vasospasm*** group compared to the ***Non-Vasospasm*** or ***Control*** groups (Table 2).

**Table 2:** Contractile response to ET-1 in rat basilar arteries with (E^+^) and without (E^−^) endothelium following incubation with human CSF.

	**E^+^**			**E^−^**		
		**Non-Vasospasm**	**Vasospasm**	**Ctrl**	**Non-Vasospas**m	**Vasospasm**
	**(n = 6)**	**(n = 6)**	**(n = 6)**	**(n = 8)**	**(n = 6)**	**(n = 6)**
***E_max1_ ± SEM*(mN/mm)**	1.04±0.13	0.86±0.11	2.10 ± 0.28*	0.81±0.08	0.83±0.15	0.92±0.07
***E_max2_ ± SEM*(mN/mm)**	2.27 ± 0.15	2.09±0.15	3.38 ± 0.12*	1.82±0.11	1.83±0.17	2.03±0.13
***pEC_50(1)_ ± SEM***	12.13 ±0.50	12.33±0.44	13.01 ± 0.36	12.3±0.51	12.0±0.49	12.01±0.28
***pEC_50(2)_ ± SEM***	9.19 ± 0.34	8.50±0.27	10.26 ± 0.37*	8.69±0.22	8.47±0.34	8.23±0.24

Values are expressed as mean ± SEM. Significance was determined by one-way ANOVA with Tukey-Kramer post-hoc where; ctrl = control

* p<0.05 = Vasospasm versus Control and Non-Vasospasm.

#### Experiments in arteries without endothelium

In E^−^ vessels all the three groups exhibited a similar biphasic response (Table 2). The enhanced contractile response to ET-1 observed in arteries with endothelium and exposed to CSF from patients with vasospasm was absent in arteries without endothelium (Fig. 2 lower panel).

ET-1 concentrations in CSF used for vessels incubation were 2.45 ±0.81 pg/ml in ***Non-Vasospasm group*** and 2.63±1.31 pg/ml in ***Vasospasm*** group. After dilution, ET-1 concentrations in the culture medium at the beginning of the incubation were undetectable in all groups (data not shown), but ET-1 concentration after 24 hours incubation was significantly higher in the ***Vasospasm*** (1.71± 0.4 pg/ml) compared to the ***Non-Vasospasm***(0.82± 0.2 pg/ml) and ***Control*** groups (0.91± 0.04 pg/ml) (p<0.05).

### Endothelin Receptor Expression in the *Ex Vivo* Model of SAH-induced Vasospasm

**Figure 3:**
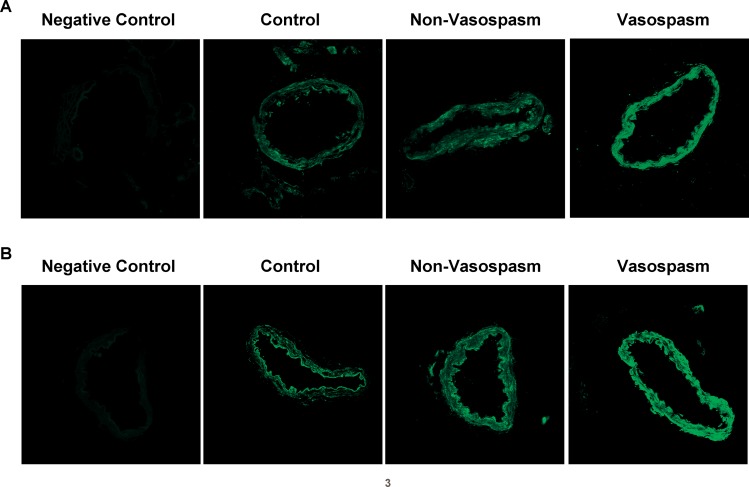
Immunoreactivity of ET Receptors. Representative examples of rat basilar arteries showing immunoreactivity in the smooth muscle cell layer of ET_A_ (panel A) and ET_B_ (panel B) following incubation with human 5% CSF from ***Control*** (n = 9), ***Non-Vasospasm*** (n = 8) and ***Vasospasm*** patients (n = 9). Negative controls for ET_A_ and ET_B_ antibodies were obtained excluding primary antibodies and in both cases resulted in no specific staining Pictures were obtained using a × 40 objective.

There was a faint autofluorescence in the arterial segments examined by confocal microscopy. When autofluorescence was subtracted ET_A_ receptors were found to be constitutively expressed in the smooth muscle cell layer of arteries exposed to CSF from all three patient groups (Fig. 3A). Semiquantitative immunofluorescence for ET_B_ receptors revealed that the expression apparently was markedly enhanced in the smooth muscle cell layer comparing arteries incubated with CSF from patients with ***Vasospasm*** than in those incubated with CSF from ***Non-Vasospasm*** and ***Control*** patients (Fig. 3B) (260±9%; p<0.001, results not shown).

### Effects of Selective and combined Endothelin Receptor Blockade

**Figure 4:**
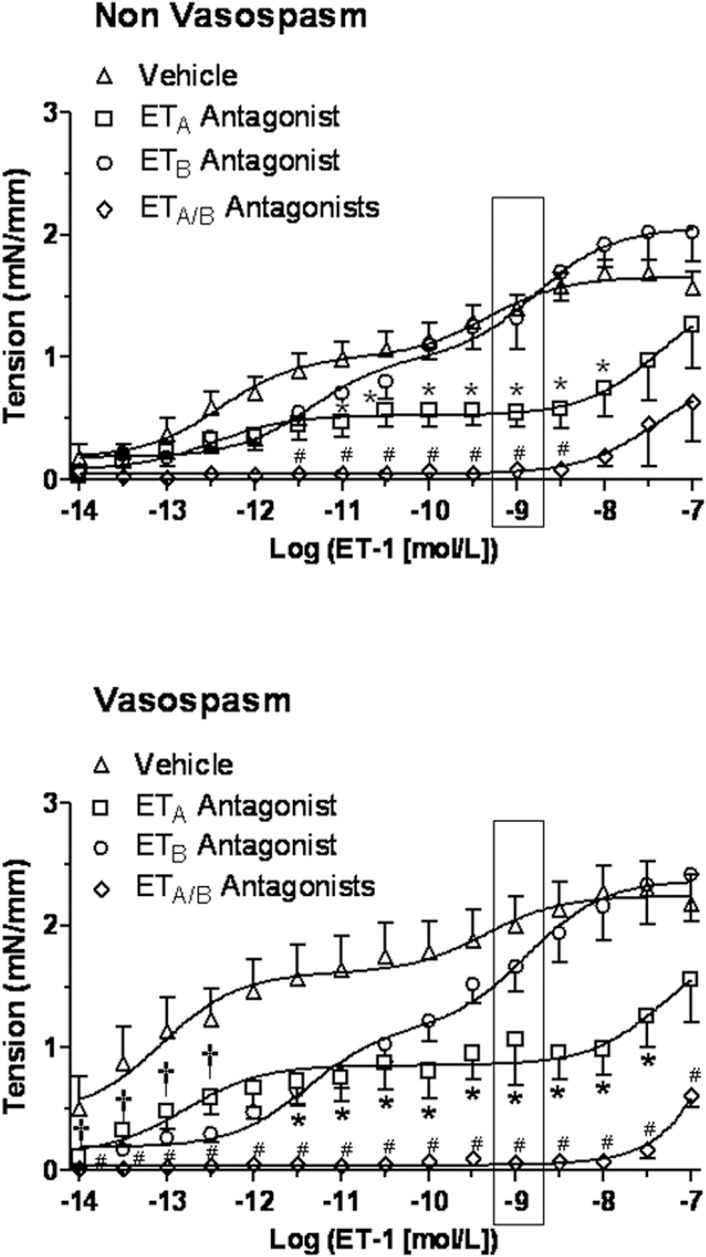
Concentration-response curves for ET-1 after ET_A_ (BQ-123), ET_B_ (BQ-788) or both receptors blockade on isolated rat basilar arteries following incubation with 5% CSF from *Non-Vasospasm* (panel A) and *Vasospasm* (panel B) patients. In the ***Non-Vasospasm*** group, the response to ET-1 was significantly reduced by the ET_A_ antagonist (n = 8), was not altered by the ET_B_ (n = 8) antagonist and greatly reduced by both antagonists (n = 5) compared to Vehicle (n = 8), whereas in the ***Vasospasm*** group, the increased sensitivity to ET-1 was significantly reduced by the ET_B_ (n = 9) antagonist in the low range, by the ET_A_ antagonist (n = 9) in the high range of ET-1 concentrations and was completely abolished by combined receptors blockade (n = 5) compared to Vehicle (n = 9). Significance at each concentration by a two-way ANOVA followed by a one-way ANOVA with Dunnett’s post-hoc analysis between groups, where *p<0.05 = ET_A_ antagonist versus vehicle, †p<0.05 = ET_B_ antagonist versus vehicle, #p<0.05 = ET_A_ and ET_B_ antagonists versus vehicle.

In a separate set of experiments, the ET-1 concentration-response curve was obtained with and without blockade of the individual and combined ET_A_ and ET_B_ receptors (Table 3). In cerebral arteries incubated with CSF from ***Non-Vasospasm*** group pretreatment with ET_A_ antagonist (BQ-123) reduced the contractile response for ET-1 concentrations between 1×10^−11^ to 1×10^−8^ mol/L; pretreatment with ET_B_ antagonist (BQ-788) did not alter ET-1 induced contraction, while pretreatment with both ET_A_ and ET_B_ antagonists abolished the contractile response for ET-1 concentrations between 1×10^−11.5^ to 1×10^−8^ mol/L (Fig. 4, upper panel). In cerebral arteries incubated with CSF from the ***Vasospasm*** group pretreatment with ET_A_ antagonist (BQ-123) significantly reduced the contractile response in the high range of ET-1 concentrations (between 1×10^−11.5^ to 1×10^−7.5^ mol/L), pretreatment with ET_B_ antagonist reduced the contractile response in the low range of ET-1 concentrations (between 1×10^−14^ to 1×10^−12^ mol/L), while pretreatment with both ET_A_ and ET_B_ antagonists completely abolished the ET-1 induced contraction (Fig. 4, lower panel).

**Table 3:** Contractile response to ET-1 of intact rat basilar arteries following incubation with human CSF and ET_A_ antagonist (BQ-123), ET_B_ antagonist (BQ-788) and combined ET_A_ and ET_B_ antagonists (BQ-123 and BQ-788).

		***Biphasic Curves***				***Sigmoidal Curves***	
		***E_max1_ ± SEM*(nN/mm)**	***E_max2_ ± SEM*(nN/mm)**	***pEC_50(1)_±SEM***	***pEC_50(2)_±SEM***	***E_max_ ± SEM*(nN/mm)**	***pEC_50_ ± SEM***
**Ctrl**	**Vehicle** (n = 7)	0.79±0.26	1.52±1.18	13.13 ± 0.71	8.67 ± 0.54		
	**BQ-123** (n = 8)					0.65 ± 0.07	14.57 ± 3.40
	**BQ-788** (n = 7)					2.17 ± 0.13	9.74 ± 0.19
	**BQ-123 + BQ-788 (n = 5)**					0.26±0.08	7.67±0.52
**NVS**	**Vehicle** (n = 8)	1.00 ± 0.09	1.65 ± 0.07	12.40 ± 0.29	9.35 ± 0.32		
	**BQ-123** (n = 8)	0.53 ± 0.19†	1.56 ± 0.51	12.49 ± 0.62	7.39 ± 0.53†		
	**BQ-788** (n = 8)	1.02 ± 0.08	2.06 ± 0.21	11.36 ± 0.35	8.79 ± 0.29		
	**BQ-123 + BQ-788 (n = 5)**					0.88±0.32	7.42±0.42
**VS**	**Vehicle** (n = 9)	1.61 ± 0.32 *	2.29 ± 0.16*	13.03 ± 0.54	9.22 ± 0.64		
	**BQ-123** (n = 9)	0.86 ± 0.43†	1.91 ± 0.82	12.81 ± 0.54	7.29 ± 0.78†		
	**BQ-788** (n = 9)	1.16 ± 0.10†	2.17 ± 0.10	11.83 ± 0.33	9.16 ± 0.31		
	**BQ-123 + BQ-788** (n = 5)					0.61±0.27	7.31±0.23

Responses were characterized by E_max_ and pEC_50_ values (negative logarithm of the molar concentration that produced half-maximum contraction). NVS = non vasospasm, VS = vasospasm. Values are expressed as mean *±* SEM. Significance was determined by a one-way ANOVA with a Tukey-Kramer post-hoc analysis between CSF, where * p<0.05 = Vasospasm versus Control and Non-Vasospasm, and by a Student’s t-test between antagonists and vehicle, where †p<0.05 = BQ-123 versus Vehicle.

## Discussion

This study for the first time clearly describes the functional response, protein expression, and effects of selective and combined blockade of both ET_A_ and ET_B_ receptors in two well-defined conditions: intact conductive cerebral vessels exposed to the biological effects of the hemorrhagic event (SAH without vasospasm) and vessels exposed to the combined biological effects of SAH and vasospasm. When CSF from patients with SAH and vasospasm was used both receptors were present and mediated the vasoconstriction; therefore combined blockade of both receptors led to the maximal inhibition of the ET-1 induced contraction. The enhanced endothelin contraction was absent in vessels without endothelium suggesting that an endothelium-dependent mechanism contribute to the enhanced contractile endothelin-1 response observed in arteries conditioned by CSF from SAH patients with vasospasm.

### Upregulation of the endothelin system after incubation with CSF from patients with vasospasm

In most functional studies on isolated vessels the two conditions of SAH, with and without vasospasm were not differentiated [6,9,10,12]. Only one study with human CSF clearly differentiated the two SAH conditions but in that study the role of endothelin-1 was not investigated[27]. The main novelty of the present study is the selection of human CSF with a definitive diagnosis of vasospasm established by angiography and integrated with TCD and clinical examination to investigate the endothelin-1 pathway in a model of isolated cerebral vessels. Interestingly, we found that intact vessels incubated with CSF from SAH patients with vasospasm resulted in a greater contractile response to ET-1 demonstrated by the left shift of the concentration response curve with higher Emax and pEC_50_ (i.e. increased potency and sensitivity to ET-1) when compared to vessels incubated with CSF from non-vasospasm and control patients. The increased sensitivity to ET-1 induced by organ culture was presumably present in all our different experimental groups and therefore equally influenced all our results. In our study cerebral arteries were incubated with Dulbecco medium containing 5% human CSF. Although ET-1 was detectable in all CSF samples of the patients studied, we applied a dilution factor which brought ET-1 concentrations below the detectable range in both groups. Therefore, the amount of ET-1 measured in the culture medium was the result of the stimulation of the arterial segments by CSF from the patients. The choice of CSF from day 4–5 for the present study is explained by the fact that we verified that patients with severe angiographic vasospasm on day 7 (**clinical vasospasm**) had a significant increase in MCA velocity on TCD on day 4–5 suggesting that molecular mechanisms responsible for vasospasm were already activated at that time. Among the soluble factors derived from the blood, oxidative stress [28], increased coagulation, fibrinolysis cascade, arachidonic acid metabolites and inflammatory mediators [29] may be involved. Recently it has been suggested that SAH induces early activation of the MEK-ERK1/2 pathway in cerebral artery walls, which is associated with upregulation of proinflammatory cytokines and MMP-9 [30].

The vessels incubated with CSF from patients with SAH and vasospasm had higher secreted ET-1 levels suggesting that soluble factors present in the CSF of this group of patients were able to trigger greater ET-1 secretion compared to CSF from patients without vasospasm. It is a limitation that only CSF obtained at day 4–5 were examined, but on the other hand that was where the vasospasm was most pronounced. Moreover, it would be an advantage if secretion and “de novo” production of ET-1 could have been discriminated by measurements of mRNA levels.

### Involvement of ET_A_ and ET_B_ receptors after incubation with CSF from patients with vasospasm

In physiological conditions the biological effects of ET-1 are mediated through activation of the two ET receptors: ET_A_ which induces vasoconstriction on smooth muscle cells, whereas the endothelial ET_B_ mediates vasodilation [31]; consequently endothelium removal leads to the abolishement of the vasodilator modulation. After SAH a different endothelium dependent effect of ET-1 may be expected and the upregulation of vascular smooth muscle ET_B_ receptor may contribute to vasoconstriction [32]. Indeed a functional interaction between ET_A_ and ET_B_ also named “cross talk” has been recently proposed to explain the limited efficacy of selective ET receptors antagonists [33].

ET_A_ receptors have previously been suggested as the main responsible for the pathogenesis of vasospasm. Thus, after SAH, ET_A_ receptor expression on smooth muscle cells was unchanged but blockade of ET_A_ receptor with clazosentan reduced the contraction to ET-1 [16]. Another two studies in SAH animal models showed upregulation of mRNA expression of the ET_A_ receptor after SAH [5,6]. In the present study, ET_A_ receptors were expressed in the smooth muscle layer of arteries exposed to CSF from all three groups of patients. These findings together with the increased functional endothelin-1 response in arteries exposed to CSF from patients with vasospasm suggest that antagonism of ET_A_ receptors would be attractive. However, ET_B_ receptors have also been suggested to play a role in the pathogenesis of vasospasm [10,34,35]. In different models of SAH increased levels of ET_B_ receptor mRNA with associated enhanced functional response to ET-1 have been shown [8,11]; in a rat model of SAH, upregulation of ET_B_ receptor, specific functional response, and mRNA and protein expression were reported [9]. Selective decrease in ET_B_ receptor-dependent relaxation [36] and reduced vasospasm after specific ET_B_ receptor antagonist [17] have also been reported. In the present study, we found that the ET_B_ receptor was not constitutively expressed in smooth muscle cells in control conditions and, more importantly, when using CSF from SAH patients *without vasospasm* but only CSF from SAH patients *with vasospasm* induced an apparently marked expression of ET_B_ receptors in the vascular smooth muscle cell layer.

Finally, we evaluated a) the functional response of intact vessels after selective and combined blockade of ET_A_ and ET_B_ receptors and b) the functional response of intact and denuded isolated vessels to CSF from SAH patients with and without vasospasm. In the ***non vasospasm*** group, only selective ET_A_ blockade induced a dose-dependent right shift of the concentration-response curve. This is explained by the fact that ET_A_ is constitutively expressed in control conditions; the hemorrhagic event on its own does not cause upregulation of ET_B_ receptors so that ET_B_ blockade had no effect. In the ***vasospasm*** group, both receptor types were upregulated. ET_B_ expression on smooth muscle cells was associated with increased tone and consequently antagonism of both receptors induced a right shift of the ET-1 concentration-response curves: the effect of ET_B_ blockade was significant at low ET-1 concentrations (high affinity phase) whereas the effect of ET_A_ blockade was significant at high ET-1 concentrations (low affinity phase). After blocking both receptors the increased tone was abolished throughout the ET-1 concentration range suggesting the involvement of both receptors in the enhanced contractile response to ET-1. Furthermore the contractile response elicited by incubation with CSF from patients with vasospasm was abolished in vessels without endothelium suggesting that the condition of vasospasm after SAH induced an alteration in endothelial function contributing to the enhanced tone. These results may be explained by 1) ET-1 secreted by endothelial cells in the culture medium contributes to vasoconstriction [37]; 2) the existence of ET_A_ receptor in the spastic vessels [7]; 3) the dual “vasodilator-vasoconstrictor” role of ET_B_ receptors as described in pulmonary vessels [38,39]; 4) the heterodimerization of ET receptors described in pulmonary hypertension [38]. The latter hypothesis may explain why combination of selective ET_A_ and ET_B_ antagonists resulted in near maximal inhibition of ET-1 induced vasoconstriction.

## Conclusion and Perspectives

What additional information does the present *translational* study provide after several clinical trials [18–20,22,40] with endothelin receptor antagonists which showed reduction in angiographic vasospasm but no improvement in measureable functional outcomes?

In a recent metanalysis Vergouwen et al [41] suggested that endothelin receptor antagonists might be effective in selective subgroups of patients. In this perspective our data indeed suggest that only a well-defined subgroup of patients who develop severe vasospasm induced expression of both receptors mediating vasoconstriction and therefore specific targeting ET_A_ receptors was not sufficient to maximally inhibit vasospasm. The limited efficacy of selective ET receptor antagonists may be due to the functional interaction between ET_A_ and ET_B_ receptors named “cross talk”.

The “cross talk” between the two receptors can be functionally interpreted as a cooperative inhibition by combined selective ET receptor blockade of the ET-1 induced contraction [33,42]. In line with this hypothesis in the present study, only blockade of both receptors induced an optimal reduction in the contractile response to ET-1.

In conclusion we studied the functional response, protein expression, and the effects of selective and combined blockade of both receptors in two well-defined conditions: intact conductive cerebral vessels exposed to the biological effects of the hemorrhagic event (SAH without vasospasm) and vessels exposed to the combined biological effects of SAH and vasospasm. When CSF from patients with SAH and vasospasm was used both receptors were upregulated and mediated vasoconstriction; therefore combined blockade of both receptors led to a maximal inhibition of ET-1-induced contraction. This enhanced ET-1 contraction was abolished in vessels without endothelium suggesting that the condition of vasospasm after SAH induced an alteration of endothelial function contributing to the enhanced tone. Further studies are required to demonstrate the exact nature of the functional interaction or “cross talk” between the two receptors [33].
